# Relationship between Masticatory Function and Bone Mineral Density in Community-Dwelling Elderly: A Cross-Sectional Study

**DOI:** 10.3390/healthcare9070845

**Published:** 2021-07-05

**Authors:** Kumi Ikebuchi, Yuhei Matsuda, Mayu Takeda, Miwako Takeda, Takafumi Abe, Kazumichi Tominaga, Shozo Yano, Minoru Isomura, Toru Nabika, Takahiro Kanno

**Affiliations:** 1 Department of Oral and Maxillofacial Surgery/Oral Care Center, Shimane University Faculty of Medicine, 89-1 Enya Cho, Izumo 693-8501, Shimane, Japan; ikebuchi@med.shimane-u.ac.jp (K.I.); yuhei@med.shimane-u.ac.jp (Y.M.); mtakeda@med.shimane-u.ac.jp (M.T.); 2 Center for Community-Based Healthcare Research and Education (CoHRE), Organization for the Promotion of Project Research, Shimane University, 223-8 Enya Cho, Izumo 693-8501, Shimane, Japan; cohre1@med.shimane-u.ac.jp (M.T.); t-abe@med.shimane-u.ac.jp (T.A.); 3 Tominaga Dental Office, 97-3 Yamada, Ohnan Cho, Ohchi 696-0313, Shimane, Japan; shika-t@ohtv.ne.jp; 4 Department of Laboratory Medicine, Shimane University Faculty of Medicine, 89-1 Enya Cho, Izumo 693-8501, Shimane, Japan; syano@med.shimane-u.ac.jp; 5 Faculty of Human Science, Shimane University, 1060 Nishikawatsu Cho, Matsue 690-8504, Shimane, Japan; isomura@hmn.shimane-u.ac.jp; 6 Department of Functional Pathology, Shimane University School of Medicine, 89-1 Enya Cho, Izumo 693-8501, Shimane, Japan; nabika@med.shimane-u.ac.jp

**Keywords:** masticatory function, bone mineral density, community-dwelling elderly, cross-sectional study, propensity score analysis

## Abstract

The relationship between masticatory function and bone mineral density (BMD) is unclear. This cross-sectional study examined this relationship after adjusting for confounding factors. The subjects were 702 community-dwelling elderly adults (306 men, 396 women) who had been recruited for the Community-Based Healthcare Research and Education study in 2019. Objective masticatory function was assessed using the gummy jelly method. The median for each descriptive statistic was 69.0 years for age, 86.2% for the young adult mean, and 18.0 for masticatory function. Comparisons of the groups with good and poor masticatory function by sex revealed a significant difference in muscle mass and the tooth number for both sexes (*p* < 0.05). Men showed significant differences in age (*p* < 0.05) and salivary occult blood findings (*p* < 0.05). Multivariate analysis using propensity scores showed a significant association between masticatory function and BMD in both sexes (men: odds ratio 163.0, 95% confidence interval 1.36–19,610.55, *p* = 0.04; women: odds ratio 48.65, 95% confidence interval 1.52–1561.15, *p* = 0.03 in women). Masticatory function and BMD in the community-dwelling elderly may be related. However, other factors, including frailty and sarcopenia, may also be involved. Regular oral health care by dentists and dental hygienists may benefit this population.

## 1. Introduction

The proportion of the Japanese population aged 65 years and older has increased from approximately 10% in 1985 to 21% in 2007, making Japan a super-aged society [1]. Along with the increasing number of older persons in Japan, a substantial increase in health care and social security problems is expected. The Japanese government has already started developing health care policies to prepare for this daunting scenario, and it has been promoting initiatives to increase the healthy life expectancy of the elderly and reduce health care costs in this age group [2,3]. Considerable attention is now focused on frailty, namely, an early mild decline in physical function [2]. Frailty is a state of vulnerability to poor resolution of homeostasis on exposure to a stressor event due to cumulative age-related deficits across multiple physiological systems [4]. Although frailty represents a physiological decline in physical function, it is under the control of various social, mental, and physical environmental factors. Frailty is considered a precursor to disability, and various triggers, such as falls, hospitalization, institutionalization, bone fractures, and cognitive decline have been shown to lead to disease or disability, which in turn, is associated with various negative health outcomes [4,5,6,7,8,9,10,11,12]. Therefore, prevention strategies, implemented in the preliminary stages of functional impairment, could be expected to have a beneficial effect.

A progressive decline in physiological reserves inevitably occurs with aging and produces several symptoms of frailty, one of which is sarcopenia, defined as age-related loss of muscle mass or strength [13,14]. Sarcopenia is usually diagnosed by measuring muscle mass in the leg or screening for grip strength and walking speed according to the criteria recommended by the Asian Working Group for Sarcopenia [15]. However, in recent years, it has become clear that sarcopenia is a state affecting the entire body, not only the lower limbs [16]. Komatsu et al. pointed out that loss of skeletal muscle mass can lead to secondary sarcopenia; particularly, loss of muscle mass related to swallowing, which in turn, may be a risk factor for aspiration pneumonia [17]. Furthermore, clinical studies in patients with stroke-induced sarcopenia suggest that oral health status may influence the severity of systemic sarcopenia [18]. The perioral and swallowing muscles are particularly affected by nutritional status and other factors [19]. To provide a more specific example, a study that assessed sarcopenia of the tongue muscles by measuring the tongue thickness found an association between nutritional status and sarcopenia of the tongue muscles [20,21]. It has been reported that the tongue muscle status affects not only swallowing function but also masticatory function [22,23]. Mastication is the process of crushing food in the oral cavity and mixing it with saliva to make it easier to swallow [24]. Masticatory function is reportedly related to a decline in the function of the perioral muscles and a decreased number of remaining teeth [25]. Therefore, it is assumed that sarcopenia and masticatory function are related.

A relationship between sarcopenia and bone mineral density (BMD) has also been suggested [26]. A prospective cohort study of the association between frailty (sarcopenia) and BMD in 235 community-dwelling women found an association of decreased physical function with decreased BMD at 1 year.

In summary, muscle weakness due to sarcopenia causes not only generalized but also localized muscle weakness, particularly in the muscles involved in feeding and mastication, such as the swallowing and occlusal muscles. At the same time, sarcopenia may cause a decrease in BMD. Although there have been reports on the relationship between BMD and sarcopenia of the tongue, the relationship between BMD and the function of the masticatory muscles remains unclear. Therefore, the aim of this study was to clarify the relationship between masticatory function and BMD in community-dwelling elderly individuals.

## 2. Materials and Methods

This study used the same dataset used in two previous cohort studies, although it had a different purpose and used different methods for analysis [27,28]. The Ethics Committee of the Shimane University Faculty of Medicine approved the study protocol in 2019 (approval number: 4570). Written informed consent was obtained from all study participants.

### 2.1. Shimane Community-Based Healthcare Research and Education (CoHRE) Study

The aim of the Shimane CoHRE study, which was designed as a cohort study, was to examine the determinants of lifestyle-related diseases, including oral health, in rural areas in the southern part of Shimane Prefecture, Japan [27,28].

### 2.2. Study Design

The participants in the present cross-sectional study were recruited in 2019 for the Shimane CoHRE study.

#### 2.2.1. Inclusion Criteria

- National Health Insurance cover
- Residents of Ohnan township
- Age > 65 years
- Participation in the 2019 survey

#### 2.2.2. Exclusion Criteria

None.

### 2.3. Collection of Data

The following data were selected at the responsibility of the investigators, and each test was applied correctly in all cases.

#### 2.3.1. Background Characteristics

We collected data for the following variables: age (years), sex, body mass index (BMI; calculated as kg/m^2^), self-reported physical activity (engaged in physical activity regularly [yes] vs. does not engage in physical activity regularly [no]), number of falls in the past year, and muscle mass.

Body composition and body weight were measured using the bioelectrical impedance analysis method with an MC-780A multi-frequency segmental body composition analyzer (Tanita Co., Tokyo, Japan) [29,30]. The muscle mass (kg) of the arms and legs and BMI were automatically calculated [31]. Body height was measured using a stadiometer.

#### 2.3.2. Oral Health Status

The number of remaining teeth was counted by a dentist or trained dental hygienist [27,32]. Salivary occult blood was evaluated using a urine occult blood test paper and a 5-point Likert scale (1, no occult blood; 5, severe disease [33,34,35]).

#### 2.3.3. Masticatory Function

Masticatory function was objectively assessed using gummy jelly (Gumi 15; soft chewy candy: Sugarless Fine Gummy, Fine Co., Ltd., Tokyo, Japan). Participants using dentures were asked to chew the gummy jelly as vigorously as possible, while wearing the denture, for 15 s to make the jelly as small as possible. Then, they were instructed to spit it into a paper cup. The number of gummy jelly pieces larger than approximately 3 mm was counted to obtain the 15-s gummy value. Based on previous research [32], the study participants were then divided into two categories; those with less than 12 gummy jelly pieces (low group) available and those with 12 or more gummy jelly pieces (high group) available.

#### 2.3.4. BMD

Bone status was measured by quantitative ultrasound (QUS) using a Benus α system (Ishikawa Seisakusho, Ltd., Ishikawa, Japan). QUS enables evaluation of bone quality, especially the microarchitecture of the calcaneus, and has the advantages of zero radiation exposure, low cost, and portability. The estimated value compared to the young adult mean (%YAM) with the same gender of examine, that is, 100% means the same value as that in healthy young men or women [28].

### 2.4. Statistical Analysis

Given that a substantial number of patients had missing data, multiple imputation using an ordinal logistic imputation method was utilized, with the assumption that the missing data were missing at random. Data normality was tested using the Shapiro–Wilk test. The following analyses were stratified by sex to eliminate definite confounding factors. For descriptive statistics, the median (interquartile range [IQR]) was calculated. The chi-square test and Mann-Whitney *U* test was used for comparisons between the low and high groups. We used propensity score analyses to balance measurable confounders between the low and high groups. Multivariable logistic regression was used to predict the number of gummy jelly pieces based on confounding covariates, including age, BMI, physical activity, number of falls, muscle mass, number of remaining teeth, and the salivary occult blood findings. Each patient was then assigned an estimated propensity score, which was the predicted probability of the number of gummy jelly pieces on the basis of his/her observed characteristics at baseline. Finally, multivariable logistic regression analyses were performed by applying propensity scores to adjust for group differences in six ways: (1) an unadjusted model; (2) a multivariable-adjusted model; (3) stratified analysis by the within-propensity score quintile; (4) regression adjustment (i.e., inclusion of the propensity score as a linear predictor in the model); (5) propensity score matching, which paired low and high groups that were similar, in terms of their measurable characteristics; and (6) use of the propensity score to create stabilized weights, defined as the inverse probability of treatment weighting [36,37,38].

All statistical analyses were performed using SPSS version 26.0 software (IBM Japan, Tokyo, Japan). Statistical significance was set at *p* < 0.05.

## 3. Results

### 3.1. Patient Demographics and Characteristics

In total, 702 participants (306 men, 396 women) were enrolled. Their characteristics are shown in Table 1. The median age was 69.0 (65.0–72.0) years for the full dataset, 70.0 (65.0–72.0) years for men, and 69.0 (65.0–72.0) years for women, with median BMI values of 22.6 (20.7–24.9), 23.0 (21.4–24.9), and 22.4 (20.1–24.8) kg/m^2^, respectively. The overall number of participants involved in physical activities was 368 (52.4%); this number included 160 (52.3%) men and 208 (52.5%) women. None of the subjects had a history of falls. For the overall study cohort, men, and women, the median muscle mass was 37.9 (34.2–46.7), 47.8 (44.3–51.7), and 34.6 (32.5–36.4) kg; the median YAM values were 86.2% (79.1–94.8%), 91.9% (84.8–100.0%), and 81.4% (76.2–89.8%); the median number of remaining teeth was 25 (16.0–28.0), 25.0 (14.4–28.0), and 25.0 (17.5–28.0); the median masticatory function values were 18.0 (11.0–25.0), 20.0 (11.0–27.0), and 17.0 (11.0–23.0); and the median salivary occult blood scores were 3.0 (1.0–4.0), 3.0 (1.0–4.0), and 3.0 (1.0–4.0), respectively.

### 3.2. Comparison of Each Variable according to Number of Gummy Jelly Pieces

Table 2 shows the results of the Mann-Whitney *U* test for masticatory function. Masticatory function was significantly associated with age, muscle mass, and number of remaining teeth in men, and with muscle mass, number of remaining teeth, and salivary occult blood findings in women However, YAM was not significantly associated with masticatory function in men or women.

### 3.3. Comparison of Bone Status according to the Odds Ratio for Masticatory Function

Table 3 shows the results of the multivariate logistic regression analysis and the results of the analysis using propensity scores. When analyzed separately for men and women, some significant differences were found in the results of stabilized inverse probability of treatment weighting for both sexes.

## 4. Discussion

The major findings of the present study suggest that there might be an association between BMD and masticatory function in healthy elderly people in Japan, especially in rural areas in the southern part of Shimane Prefecture. A search of the past literature suggested that this association is more likely to be indirect than direct.

Our study was conducted in rural rather than urban areas in Japan and focused on the Ohnan area, where the super-aged society is the most advanced. According to their median age, the study participants would be considered early elderly in Japan, who are relatively young [39]. The target population, both male and female, exhibited normal weight (BMI 18.5–24.9 kg/m^2^) when evaluated using the World Health Organization classification [40]. A discrepancy has been found between the self-reported number of remaining teeth and the number determined on examination by dental professionals [41]. However, the data in this study were obtained from examinations by dentists and dental hygienists, so it can be considered reliable. According to a report by Matsui et al., the average number of remaining teeth in the age group included in this study is 24.9–24.2. Therefore, the number of remaining teeth in our participants was above the reported average [41], and the participants were considered to have good occlusal status and masticatory function, as observed in a study by our research team four years ago [32]. Furthermore, none of the subjects had experienced falls, and more than half engaged in daily exercise, which suggests that it was a highly physically active population. The salivary occult blood test is rarely used internationally, and its reliability, validity, and accuracy when used to screen for periodontal disease are debatable. However, in Japan, it is often used as an indicator in mass examinations and can roughly estimate the presence or severity of periodontal disease [42]. The high prevalence of salivary occult blood in the study population is reasonable, given that periodontal disease is very common, affecting up to 90% of the population worldwide [43]. Therefore, our target population can be considered a group of healthy elderly people who can come for checkups, and we believe our findings can be generalized to the healthy elderly population living in rural areas.

In this study, we found a significant difference in muscle mass when comparing masticatory function between men and women. A review by Hatta et al. reported that occlusal force and tongue pressure were associated with sarcopenia, indicating a decrease in the total body skeletal muscle mass, and showed similar results [44]. Masticatory function is related to the number of remaining teeth, and our study results are consistent with those of a previous report that also included both men and women [45]. One parameter that showed a significant difference specifically in the female population was the salivary occult blood score. To the best of our knowledge, there are no reports on sex as a risk factor for periodontal disease. However, a review article has pointed out that the risk of periodontal disease is increased in women with osteoporosis after menopause [46]. Therefore, the salivary occult blood score could be higher for women. Our present findings suggest that if men had the same level of periodontal disease because of fewer teeth, women with more teeth and more periodontal pockets could have more occult blood in their saliva. Therefore, we consider that our target population of general community-dwelling healthy elderly individuals had background factors similar to those already characterized in the literature.

**Figure 1:**
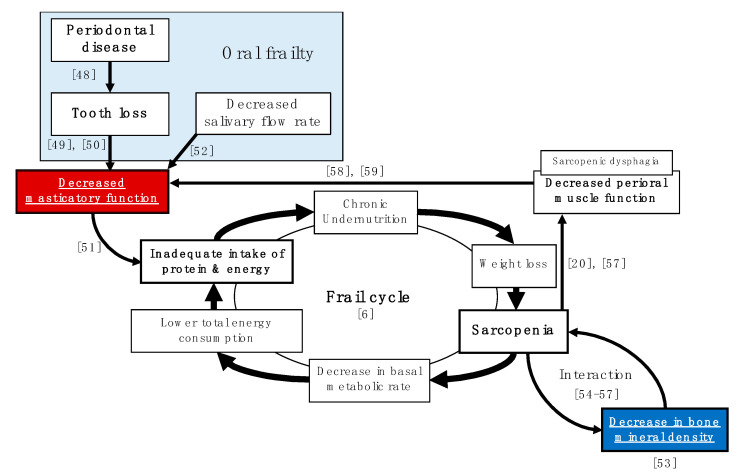
Pathways for the relationship between bone mineral density loss and masticatory function loss that promote the flail cycle. (Numbers in the figure indicate references).

In the analysis, with adjustment for confounding by propensity score, there was a significant association between masticatory function and BMD in both men and women. Our results indicate that there is a relationship between masticatory function and BMD, regardless of sex. Figure 1 shows that the relationship between BMD and masticatory function may be related to multiple indirect factors rather than just one direct factor. Undernutrition not only leads to a localized decline in nutritional indices, but also affects the entire body, leading to weight loss, sarcopenia, a lower resting metabolic rate, decreased total energy expenditure, inadequate protein and energy intake, and chronic undernutrition, which results in a flail cycle [5]. In this flail cycle, the decrease in BMD and the decline in masticatory function may act independently as accelerators. The decline in masticatory function is mainly caused by tooth loss due to periodontal disease and decreased salivary gland function, which in turn, leads to inadequate intake of protein and energy [24,47,48,49,50,51]. It has also been reported that BMD loss and sarcopenia caused by physiological factors can interact with and exacerbate each other [52,53,54,55]. Moreover, sarcopenia has been suggested as a possible accelerator of the decline in masticatory function. Sarcopenia has also been reported to cause sarcopenic dysphagia, which involves loss of function of the perioral muscles [19,56], particularly the masticatory muscles (temporal, masseter, lateral pterygoid, and medial pterygoid muscles), and consequently leads to decreased masticatory function [57,58].

In Figure 1, there are only a limited number of factors, and improving masticatory function, in particular, is a strategy that can be easily addressed by prosthetic dentistry [59]. In recent years, attention has also been focused on oral frailty, which appears to be a preliminary symptom of general frailty. Oral frailty is a prelude to a decline in oral function and can be reversed by paying attention to daily oral health care. Therefore, it is important to maintain a high level of oral literacy on a daily basis, even before tooth loss and decline in oral function occur by ensuring contact with a family dentist and regular oral health management by a dentist or dental hygienist.

This study has several limitations. First, it did not adjust for confounding factors, such as the use of bone-modifying drugs, including bisphosphonates and molecular targeted drugs, use of hormone therapy, menopausal status, reasons for tooth loss, and masticatory units. Second, given that BMD was measured by indirect methods, it may not accurately reflect the presence or absence of osteoporosis. Third, the study had a cross-sectional design; therefore, the causal relationship remains clear. Fourth, we did not incorporate most of the relevant factors underlying the mechanisms, shown in Figure 1, as survey items. Fifth, the statistical analysis was not sufficiently robust because of the statistical methods used. In future, a large-scale longitudinal cohort study that includes a wide range of factors related to frailty in the survey items is necessary to determine the relationship between masticatory function and BMD in more detail.

## 5. Conclusions

The findings of this study suggest that masticatory function and BMD in healthy elderly people living in the community may be related. However, they also suggest that many complex factors may be involved in this relationship. Finally, our findings indicate that regular oral health care by dentists and dental hygienists may play an important role in this population.

## Figures and Tables

**Table 1:** Demographic data.

Variable	Full Dataset (n = 702)	Men (n = 306)	Women (n = 396)
Age (years)	69.0 (65.0–72.0)	70.0 (65.0–72.0)	69.0 (65.0–72.0)
Body mass index (kg/m^2^)	22.6 (20.7–24.9)	23.0 (21.4–24.9)	22.4 (20.1–24.8)
Physical activity (yes)	368 (52.4)	160 (52.3)	208 (52.5)
Falls, n	0.0 (0.0–0.0)	0.0 (0.0–0.0)	0.0 (0.0–0.0)
Muscle mass (kg)	37.9 (34.2–46.7)	47.8 (44.3–51.7)	34.6 (32.5–36.4)
Bone status YAM (%)	86.2 (79.1–94.8)	91.9 (84.8–100.0)	81.4 (76.2–89.8)
Remaining teeth, n	25.0 (16.0–28.0)	25.0 (14.4–28.0)	25.0 (17.5–28.0)
Masticatory function	18.0 (11.0–25.0)	20.0 (11.0–27.0)	17.0 (11.0–23.0)
Salivary occult blood result	3.0 (1.0–4.0)	3.0 (1.0–4.0)	3.0 (1.0–4.0)

The data are presented as the median (interquartile range) or number (%) for “Physical activity (yes)”. YAM, young adult mean.

**Table 2:** Comparison of each variable according to number of gummy jelly pieces.

Variable	Men			Women		
Gumi 15	<12 (n = 96)	≥12 (n = 210)	*p*-Value	<12 (n = 153)	≥12 (n = 243)	*p*-Value
Age (years)	70.0 (68.0–72.0)	69.0 (64.8–71.3)	0.02 *	69.0 (66.0–72.0)	69.0 (64.0–71.0)	0.29
Body mass index (kg/m^2^)	23.3 (21.7–25.0)	22.9 (21.3–24.9)	0.47	22.4 (20.2–25.1)	22.3 (20.1–24.6)	0.59
Physical activity (yes)	54.0 (56.3)	106 (50.5)	0.39	78 (51.0)	130 (53.5)	0.68
Falls, n	0.0 (0.0–0.0)	0.0 (0.0–0.0)	0.41	0.0 (0.0–0.0)	0.0 (0.0–0.0)	0.26
Muscle mass (kg)	46.0 (43.6–49.6)	48.3 (44.8–52.4)	<0.01 *	34.1 (32.1–35.8)	35.0 (32.9–36.6)	<0.01 *
Bone YAM (%)	90.5 (82.5–98.5)	92.6 (85.8–100.0)	0.10	80.5 (75.6–89.1)	82.0 (76.8–90.4)	0.09
Remaining teeth, n	12.0 (6.0–17.7)	27.0 (23.8–28.0)	<0.01 *	16.3 (9.0–24.0)	27.0 (24.0–28.0)	<0.01 *
Salivary occult blood result	3.0 (1.0–4.0)	3.0 (2.0–4.0)	0.07	2.0 (1.0–4.0)	3.0 (2.0–4.0)	<0.01 *

The data are presented as the median (interquartile range) or number (%) for “Physical activity (yes”). YAM, young adult mean. *: *p* < 0.05.

**Table 3:** Comparison of odds ratios for bone status according to number of gummy jelly pieces.

Variable	Men		Women	
	Odds Ratio (95% CI)	*p*-Value	Odds Ratio (95% CI)	*p*-Value
Unadjusted model	1.02 (0.99–1.04)	0.15	1.02 (0.99–1.04)	0.11
Multivariable-adjusted model	1.01 (0.98–1.04)	0.49	1.01 (0.99–1.04)	0.30
Propensity score-adjusted model				
Stratification	1.01 (0.95–1.08)	0.51	1.04 (0.97–1.10)	0.35
Within-propensity score quintile				
1 (Lowest propensity)	1.05 (0.97–1.14)	0.23	1.02 (0.96–1.09)	0.52
2	1.01 (0.95–1.07)	0.74	1.07 (0.98–1.15)	0.12
3	1.03 (0.97–1.10)	0.36	1.00 (0.94–1.05)	0.91
4	0.97 (0.90–1.05)	0.44	1.04 (0.99–1.09)	0.07
5 (Highest propensity)	1.01 (0.96–1.06)	0.76	1.05 (0.99–1.10)	0.11
Regression adjustment	1.01 (0.98–1.04)	0.62	1.01 (0.99–1.04)	0.32
Weighting (stabilized IPTW)	163.00 (1.36–19,610.55)	0.04 *	48.65 (1.52–1561.15)	0.03 *
Matching 1:1 (108 men, 288 women)	1.00 (0.97–1.03)	0.98	1.01 (0.99–1.03)	0.16

CI, confidence interval; IPTW, inverse probability of treatment weighting. *: *p* < 0.05.
